# The midterm results of coronary endarterectomy in patients with diffuse coronary artery disease

**DOI:** 10.1186/s13019-018-0776-8

**Published:** 2018-07-24

**Authors:** Zhibing Qiu, L. Auchoybur Merveesh, Yueyue Xu, Yinshuo Jiang, Liming Wang, Ming Xu, Fei Xiang, Xin Chen

**Affiliations:** 1 Department of Thoracic and Cardiovascular Surgery. Nanjing First Hospital, Nanjing Medical University, Changle Rd 68, Nanjing, 210006 Jiangsu China; 2 Department of Cardiothoracic and vascular Surgery, Nanjing First Hospital, Nanjing Medical University, 68 Changle Rd, Nanjing, 210006 China

**Keywords:** Coronary endarterectomy, Coronary artery bypass grafting, Graft patency, Follow-up results

## Abstract

**Background:**

Diffuse coronary artery disease is a challenge for both percutaneous coronary intervention (PCI) and coronary artery bypass grafting (CABG). Coronary artery endarterectomy (CE) coupled with CABG is an alternative method to achieve complete revascularization. The mid- and long-term results of CE are largely questionable. The aim is to evaluate the early and mid-term graft patency of concomitant coronary artery endarterectomy and CABG.

**Methods:**

A total of 304 patients who had undergone concomitant CE and CABG for diffuse coronary artery disease were identified from our database. A total of 238 patients (1) with complete operative records, (2) with good graft flow during surgery, (3) who were discharged, (4) with a one-year/ three-year follow-up were included in our study. The follow-up information was obtained directly from our out-patient department and by telephone contact. The categorical and continuous values were analyzed by Chi Square test and student’s test respectively.

**Results:**

CE was performed on 238 patients who represented a total of 269 target coronary vessels. The mean age of the patients was 67.8 ± 6.8 years old; male to female patient ratio was 170:68. The mean intensive care unit stay was 1.7 ± 8 days, and mean post-operative length of hospital stay was 11 ± 3 days. The average follow up time was 41.8 ± 21.4 months. At follow-up, the overall graft patency was 78.4% at one year and 69.8% at three years. The left coronary graft patency rate was significantly higher than the right coronary graft patency rate (87.4% vs 73.1% at one-year and 78.2% vs 64.8% at three years). There was no significant difference in graft patency rates between the on-pump CE + CABG vs off-pump CE + CABG groups at one year (80.0% vs 76.9%) and at three years (92.3% vs 91.7%). At the one-year follow up, 92.3% of grafts showed grade A patency in the on-pump group versus 91.7% in the off-pump group; 7.7% of grafts showed grade B patency in the on-pump group versus 8.3% in the off-pump group. At the three-year follow up, 80.6% of grafts showed grade A patency in the on-pump group versus 77.4% in the off-pump group; 19.4% of grafts showed grade B patency in the on-pump group versus 22.6% in the off -pump group. The Predictors of better graft patency are use of LIMA graft, CE on LAD, and intra-operative graft flow meter and PI.

**Conclusions:**

In patients with diffuse coronary disease, CE is a safe and feasible technique for a select group of patients with excellent mid-term survival rates and graft patency rates. CE produces better overall results when performed on the LAD, and grafted over with the LIMA. Similar outcomes are obtained with both on-pump and off-pump surgery. For a select group of patients, coronary endarterectomy (CE) offers an alternative choice of coronary artery reconstruction and complete coronary revascularization.

## Background

Revascularization of diffuse coronary artery disease is still challenging for both percutaneous coronary intervention (PCI) and coronary artery bypass grafting (CABG). These techniques often prove to be inadequate when dealing with severe diffuse coronary atherosclerosis. An alternative to achieve complete revascularization is coronary endarterectomy (CE) which was introduced in the 1950s with the first procedures performed without cardiopulmonary bypass [1]. Initially, CE followed by CABG lost popularity due to higher associated rates of perioperative morbidity and mortality as compared to CABG alone [2]. Moreover, the complications are even more fatal when CE is performed on arteries that are crucial to achieve optimal effect, such as the left anterior descending (LAD) artery, where failure to obtain optimal blood flow results in catastrophic clinical outcomes. It is debatable, however, that the difference in favorable outcomes between CE + CABG and CABG alone is due to lesion complexity. Fukui T et al., postulate that the higher risks associated with CE are due to the profile of patients with diffuse coronary vessel lesions which makes grafting challenging due to the unavailability of a soft region [3]. Data from the last 15 years report better survival rates after CE [4–6]. Many surgeons are still reluctant to perform CE due to the absence of guidelines and varying outcomes from across centers [7, 8]. The aim of this retrospective study is to evaluate the mid-term results of coronary endarterectomy followed by coronary artery bypass grafting (CE + CABG).

## Methods

### Study design

A retrospective analysis was performed on 238 consecutive patients who underwent CE + CABG at our center. This study was approved by the institutional review board of Nanjing First Hospital, Nanjing Medical University in compliance with health insurance portability and accountability act regulations and the declaration of Helsinki.

### Patient population

The data of 304 patients who underwent CE and CABG at our center under the same surgeon from January 2010 to January 2017 was reviewed. Of these, 78%(238 patients) who (1)had complete operative records, (2)good graft flow during surgery, (3) were successfully discharged, and (4) were followed up at the one-year mark were included in this study. Our study was commissioned and approved by the ethical commission of Nanjing first hospital. The patients were informed of our treatment and follow-up protocol, and written consent was obtained from all of them. The decision to perform endarterectomy was based on the preoperative angiograms and intra-operative findings. Most of the CE were scheduled before the operation, but the final decision as to use which strategy of revascularization and technique of CE was made during the operation as per the surgeon’s preference.

### Surgical procedure

All patients underwent routine median sternotomy. On pump CABG surgery was performed on 146 patients (61.3%), while the remaining underwent off-pump CABG surgery with 92 patients(38.7%). The decision to carry-out CE was made pre-operatively from the coronary artery angiographic results and intra-operatively according to the length of stenosed segment and availability of graftable regions on the calcified coronaries. CE was performed if the target coronary artery had diffuse disease with severe calcifications, and occluded or semi-occluded lumen distal to the proximal stenosed segment. A 10-20 mm longitudinal incision was made near the proximal stenosed end. Using coronary forceps, the calcified intima was gently pulled out while the coronary adventitia was simultaneously pushed in the opposite direction (closed endarterectomy). The procedure was repeated until all calcified segments were removed. Alternatively, we also used longer arteriotomies for direct vision removal of the calcified segment (open endarterectomy). The coronaries were flushed to remove any debris. Subsequently, CABG was performed. We used 3 methods of anastomosis, namely, the (1)on-lay LIMA graft to the LAD, (2) saphenous vein patch + LIMA graft to the LAD, and (3) on-lay saphenous vein graft to other territories. Before chest closure, the graft flow rate and PI value were measured and recorded using Medi-Stim Butterfly flowmeter (Medi-Stim As, Oslo, Norway).

### Study endpoints and follow-up

The primary endpoint of this study is post-procedural graft patency at one-year and three-year follow up respectively. Graft patency were classified as 1) Grade A: excellent graft patency, < 50% stenosis, 2) Grade B: graft stenosis> 50%, and 3) Grade O: total graft occlusion. The patients underwent either a coronary angiography (CAG) or a computer tomography angiogram (CTA) to assess the degree of in-graft stenosis. The secondary end-point was the incidence of major adverse cardiac and cerebrovascular events (MACCE), defined as all-cause death, non-fatal myocardial infarction and cerebrovascular event during the 3-year clinical follow-up. Follow-up data was obtained from the patients by telephone contact after surgery and/or follow-up at the out-patient department.

### Statistical analysis

Continuous data were expressed as mean ± standard deviation or median with the interquartile range and categorical data as percentages. Cumulative survival was evaluated with the Kaplan–Meier method. All reported *P* values are two-sided, and *P* values of < 0.05 were considered to indicate statistical significance. All statistical analyses were performed with SPSS 22.0 (SPSS, Inc., Chicago, IL, USA). All statistical analyses were performed with the assistance of a departmental statistician. Categorical variables were compared using either Pearson’s chi-square or Fisher’s exact tests and are expressed as a percentage of the group of origin.

Multiple variable models were constructed to determine independent factors influencing the following outcomes: advanced age, diabetes mellitus, dyslipidaemia, off-pump surgery, LIMA graft, CE on LAD and Intraoperative graft flow-meter and PI.

## Results

A total of 238 patients underwent CE + CABG during the study period. The mean age of the patients was 65 ± 8 years in the on-pump group and 63 ± 9 years in the off-pump group; the male to female ratio was 201:37. 59.8% of patients undergoing off-pump surgery had a high lipid profile (vs 43.8% on-pump), 14.1% had suffered from previous cerebrovascular accident (vs 2.1% on-pump), 23.9% suffered from concomitant COPD (vs 4.1% on-pump), 48.9% had a calcified ascending aorta (vs 2.7% on-pump), 96.7% had RCA critical stenosis > 90% (vs 55.5% on-pump). We preferred off-pump CE + CABG in patients with known hyperlipidemia, previous cerebrovascular accidents, COPD, calcified ascending aorta, and RCA critical stenosis> 90. The complete pre-operative patient characteristics are summarized in Table 1. Our patient cohort represented 269 target coronary lesions, of which 108 (40.1%) were located on the left anterior descending (LAD) artery and sub-branches, 140 (52%) were located on the right coronary artery (RCA) and sub-branches, and 21 (8.8%) were located on the left circumflex artery and/or obtuse marginal artery (LCX/OM). The mean number of bypasses performed was 4.0 ± 0.9 (on-pump) vs 3.8 ± 0.7 (off-pump), 8.9% of on-pump patients required intra-aortic balloon pump (vs 1.1% in the off-pump group). The left internal mammary artery was used in all of the patients, saphenous vein grafts were used in more than 90% of the patients, and the radial artery was used in one-fifth of the patients (see Table 2: operative characteristics). The mean graft flow rates measured intra-operatively after endarterectomy and bypass grafting was 36 ± 8 ml/min with a mean PI value of 3.1 ± 0.8.

**Table 1:** Preoperative Patient Characteristics in CE

Variable	Group on-pump (*n* = 146)	Group off-pump (*n* = 92)	*P* Value
Clinical demographics			
Age (y)	65 ± 8	63 ± 9	0.0749
Female sex	25 (17.1%)	12(13.0%)	0.398
Coronary risk factors			
Hypertension	95(65.1%)	59(64.1%)	0.883
Diabetes	122(83.6%)	76(82.6%)	0.080
Hyperlipidemia	64(43.8%)	55(59.8%)	**0.017**
Smoking	67(45.9%)	41(44.6%)	0.842
Peripheral vascular disease	28(19.2%)	15(16.6%)	0.575
Previous renal impairment	8(5.5%)	4(4.3%)	0.698
Cerebrovascular accident	3(2.1%)	13(14.1%)	**0.000**
COPD	6(4.1%)	22(23.9%)	**0.000**
Calcified ascending aorta	4(2.7%)	45(48.9%)	**0.000**
Cardiac profile			
Previous myocardial infarction	93(63.7%)	55(62.2%)	0.544
Unstable angina	106(72.6%)	68(73.9%)	0.824
previous PCI	23(15.8%)	15(16.3%)	0.910
Mean ejection fraction	0.58 ± 0.12	0.56 ± 0.11	0.1974
Poor ejection fraction(<0.35)	15(10.3%)	9(9.8%)	0.902
Left main disease	29(19.8%)	17(18.5%)	0.792
RCA Critical stenosis > 90%	81(55.5%)	89(96.7%)	**0.000**
Number of diseased vessels	3.5 ± 0.5	3.4 ± 0.6	0.1689

*COPD* chronic obstructive pulmonary disease, *PCI* percutaneous coronary intervention, *RCA* right coronary artery

**Table 2:** Operative Characteristics

Variable	Group on-pump (*n* = 146)	Group off-pump (*n* = 92)	*P* Value
Length of the ateriotomy	14.5 ± 2.5	14.1 ± 2.8	0.2525
Operative time (min)	253 ± 42	260 ± 48	0.2375
The number of distal anastomoses	4.0 ± 0.9	3.8 ± 0.7	0.0711
Associatied procedure	38(26.0%)	26(28.3%)	0.705
Intra-aortic balloon pump	13(8.9%)	1(1.1%)	**0.013**
Conduit to anastomosis vessel			
LIMA	146(100%)	92(100%)	1.000
Radial artery	29(19.9%)	19(20.7%)	0.883
Saphenous vein	138(94.5%)	88(95.7%)	0.698

*LIMA* Left internal mammary artery

The mean intensive care unit stay was 2.5 ± 0.5 days for the patients who underwent on-pump surgery and 2.6 ± 0.7 days for the patients who underwent off-pump surgery. There was no significant difference in preoperative death and MACCE rates between the two groups. The occurrence of ventricular arrhythmia was significantly greater in the off-pump group (14.1% vs 4.8% in the on-pump group) (Table 3: perioperative mortality and morbidity). The average follow up time was 41.8 ± 21.4 months. Follow-up on the patients postoperatively showed a significant reduction in angina and dyspnea across the entire cohort as compared to the pre-operative period (*P* < 0.001). Six patients died during the 3-year follow-up period, two of whom died of neoplasms, three of cerebrovascular events and one died of unknown reasons and was considered MACCE-related for statistical purposes.

**Table 3:** Perioperative Mortality and Morbidity

Variable	Group on-pump (*n* = 146)	Group off-pump (*n* = 92)	*P* Value
Death (30-day)	2(1.4%)	1(1.1%)	1.000
Perioperative myocardial infarction	6(4.1%)	3(5.4%)	1.000
Ventricular arrhythmia	7(4.8%)	13(14.1%)	**0.011**
Re-exploration for bleeding	2(1.4%)	1(1.1%)	1.000
Cerebral vascular accident	1(0.7%)	1(1.1%)	1.000
Acute renal failure	7(4.8%)	5(5.4%)	1.000
Intensive care unit stay(days)	2.5 ± 0.5	2.6 ± 0.7	0.2005
Infection	4(2.7%)	2(2.2%)	1.000

At the one-year follow-up, data of 208 (232 endarterectomized target vessels; 88.5% of the original cohort) patients were obtained. The overall graft patency was 78.4% at 1 year and 69.8% at 3 years. The left coronary graft patency rate was significantly higher than the right coronary graft patency rate (87.4% vs 73.1% at one-year, *p* = 0.011 and 78.2% vs 64.8% at 3 years, *p* = 0.032) (Table 4: follow up graft patency analysis of coronary endarterectomy). There was no significant difference in graft patency rates between the on-pump CE + CABG vs off-pump CE + CABG groups at 1 year (80.0% vs 76.9%; *p* = 0.599) and at 3 years (92.3% vs 91.7%; *p* = 0.884). At the one-year follow up, 92.3% of grafts showed grade A patency in the on-pump group versus 91.7% in the off-pump group (p = 0.884); 7.7% of grafts showed grade B patency in the on-pump group versus 8.3% in the off-pump group (*p* = 0.988). At the three-year follow up, 80.6% of grafts showed grade A patency in the on-pump group versus 77.4% in the off-pump group (*p* = 0.636); 19.4% of grafts showed grade B patency in the on-pump group versus 22.6% in the off -pump group (*p* = 0.636); (see: Table 5. Follow-up graft patency analysis between group on-pump and off-pump CE).

**Table 4:** Follow-up graft patency analysis of coronary endarterectomy

Follow-up time/patency rate	Total patency rate	Left coronary graft patency rate	Right coronary graft patency rate	*P* value
One year	78.4%(182/232)	87.4%(76/87)	73.1%(106/145)	0.011
Three year	69.8%(162/232)	78.2%(68/87)	64.8%(94/145)	0.032

**Table 5:** Follow-up graft patency analysis between group on-pump and off-pump CE

Variable	Group on-pump (*n* = 130)	Group off-pump (*n* = 78)	*P* Value
Early patency rate(1 year)	80.0%(104/130)	76.9%(60/78)	0.599
Grade A	92.3%(96/104)	91.7%(55/60)	0.884
Grade B	7.7%(8/104)	8.3%(5/60)	0.988
Midterm patency rate(3 year)	71.5%(93/130)	67.9%(53/78)	0.584
Grade A	80.6%(75/93)	77.4%(41/53)	0.636
Grade B	19.4%(18/93)	22.6%(12/53)	0.636

Grade A stands for excellent graft patency, Grade B for graft stenosis of greater than 50%, and Grade O for occlusion

**Fig. 1:**
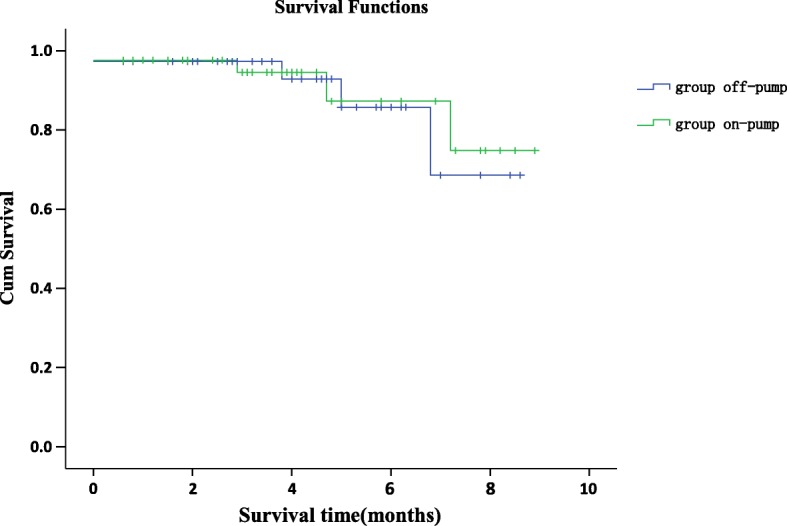
Mid-long term survival with group on-pump versus group off-pump after coronary endarterectomy

Moreover, the predictors of better graft patency are use of LIMA graft (*P* = 0.021), CE on LAD (*P* = 0.013), and intra-operative graft flow meter and PI (*P* = 0.002)(Table 6). Kaplan-Meier survival estimates in the on-pump CE + CABG group at one, 3 and 5 years were 0.96, 0.89 and 0.73 respectively and 0.95, 0.88 and 0.71 respectively in the off-pump CE + CABG group (Fig. 1: Mid-long term survival with group on-pump versus group off-pump after coronary endarterectomy).

**Table 6:** Predictors of better graft patency during follow-up

Characteristics	Odds ratio	95% CI	*P*-value
Advanced age	5.7	0.71–49.3	0.102
Diabetes mellitus	0.91	0.85–0.997	0.12
Dyslipidaemia	0.9	0.86–1.059	0.82
Off-pump surgery	1.125	0.98–1.356	0.056
LIMA graft	1.15	1.31–105.3	**0.021**
CE on LAD	23.8	1.29–46.9	**0.013**
Intraoperative graft flow-meter and PI	1.29	1.09–1.53	**0.002**

*LIMA* left internal mammary artery, *CE* coronary endarterectomy, *LAD* left anterior descending, *PI* pulsation index

## Discussion

Before the widespread use of CABG to treat coronary artery disease, CE was the mainstay treatment for improving blood flow to severely stenosed coronary arteries. Although the technique was introduced more than five decades ago, it rapidly fell out of usage [1]. Acute post-operative MI, a key complication following CE, might be associated to endothelial damage during the procedure and subsequent thrombosis. Intact coronary endothelium produces vasoactive factors to neutralize leukocyte adhesion and platelet aggregation, consequently reducing inflammation and thrombosis in blood vessels [9]. Furthermore, MI caused by residual lesion-induced occlusion also contribute to the postoperative morbidity and mortality of CE [10, 11].

The aim of endarterectomy is to extract the calcified intima distal to the incision and ensure adequate revascularization. Distal intimal extraction is considered adequate if obvious bleeding of the distal vascular segment and a patent vessel with normal distal intima (with thin and soft tissues) on the top is observed after endarterectomy. There are two methods of performing endarterectomy, namely the ‘open’ and ‘closed’ techniques [12, 13]. The decision to use either ‘open’ or ‘closed’ CE was by the operating surgeon using the following criteria: 1) type of occlusion (complete total occlusion, or > 50% occlusion, or > 75% occlusion), 2) length of stenosed segment, 3) location of the lesion (medial or distal), and 4)number of bypasses to be performed. In hospital-mortality, defined as death due to any cause within 30 days of the operation, was 1.3% for both groups. 3.8% of the patients developed myocardial infarction during the peri-operative period, and 8.4% developed ventricular arrhythmias. During the three-year follow-up period, six deaths occurred, of which four were considered to be MACCE-related (1.7%). A 2015 meta-analysis performed by Kirill LK et al. show a 2.07% mortality at 30 days, 6.0% at 1 year, and 12.10% at 3 years post CABG surgery. Although the authors predominantly investigate outcomes in an elderly patient cohort (> 50% of patients were > 70 years old), we consider 1.3% mortality rate at 30-days, and a 3-year MACCE rate of 1.7% to be acceptable for concomitant CE and CABG. At the very least, it indicates that CE doesn’t significantly increase the risk margin of the procedure [14].

As mentioned above, the patient cohort undergoing CE have a higher risk profile and the surgery itself is technically more challenging. For on-pump CE + CABG, this invariably translates to longer aortic cross-clamp, CPB times; and a greater incidence of intra-operative intra-aortic balloon pump implantation as compared to off-pump CABG (8.9% vs 1.1% respectively). We also performed off-pump CE + CABG on 38.7% of our cohort. In our study, we found that patients with a certain profile are more likely to be referred for off-pump surgery instead of on-pump surgery. Some patient characteristics include younger age, previous cerebrovascular accident, COPD, and calcified ascending aorta. One drawback of off-pump surgery is that it significantly increases the rate of peri-operative ventricular arrhythmias. There was no significant difference in early and late patency rates between the on-pump and off-pump CE + CABG groups. Off-pump CABG has beneficial effects on the recovery of the patient in the early post-operative period, as it decreases the operative time, and lowers the rate of MI, reduces the duration of inotropic support and ICU stay, and decreases the rate of peri-operative bleeding [15]. Our analysis on the on-pump and off-pump groups, show no significant difference in one-year and three-year graft patency rates; The results are similar to the study performed by Li S et al., where they also found no significant difference in survival at one-year between on-pump CABG and off-pump CABG [16]. Kaplan-Meier survival estimates at one-, three-, and five-years do not show a significant difference between the on-pump and off-pump CABG groups. It appears that the choice of either off-pump or on-pump in the setting of CE + CABG does not have deleterious effects on the early and mid-term outcomes of the procedure. In our study cohort, the mean discharge time after transfer to the ICU was (11 ± 3) days vs the 9.01 days and 7.7 days, reported by LaPar et al. for patients undergoing CE + CABG and CABG respectively; Nevertheless, we report lower mortality rates (1.42% vs 4.0%) despite our longer follow-up time (41.8 ± 21.4 months vs 27.7 ± 17.7 months) [17].

Complete revascularization is accompanied by added benefits such as ischemia reduction, improvement of LV function, reduction of arrhythmias, and preserved ejection fraction [17]. Moreover, the composite endpoint of death/MI/stroke is significantly more likely with higher degrees of incomplete revascularization [18]. The 2011 ACC/AHA guideline for coronary artery bypass graft surgery and the 2014 ESC/EACTS guideline on myocardial revascularization have no reference to the clinical recommendation of CE due to the absence of randomized controlled trials [19–21]. Despite a lack of guidelines for the feasibility of complete or incomplete revascularization in the case of concomitant CE + CABG, we decided that a strategy of complete revascularization would better fit the high risk profile of our patient cohort. We obtained satisfactory results while keeping a high number of distal anastomoses (4.0 ± 0.9 vs 3.8 ± 0.7 for on-pump vs off-pump group respectively), thus ensuring a graft vessel to each perfusion territory of myocardium.

Another significant result from our study, is the higher patency of the left coronary graft at both one and 3 years, as compared to the right coronary graft (87.4% vs 73.1%, *p* = 0.011 at one year; 78.2% vs 64.8%, *p* = 0.032 at 3 years respectively). Since all endarterectomies and bypasses were performed by the same surgeon (CX) to assure consistency of technique, this suggests that the use of LIMA graft is an independent factor accounting for the superior patency rates [22]. The endothelium of internal mammary arteries produce vasodilators, are better protected from atherosclerosis, and due to postsurgical blood flow remodeling of the graft vessel, are better conduits for bypass as compared to the saphenous vein. The LIMA graft is also a predictor for better graft patency during follow-up (*P* = 0.021) [23, 24]. Other predictors of graft patency at follow up are presence of using LIMA graft, performing coronary endarterectomy on the LAD, and good intra-operative flow and PI values. The presence of the above mentioned factors, are highly indicative of successful CE which translates into good graft patency, and hence, can be used to score a CE procedure.

### Limitations

The main limitation of this study is its retrospective nature. This is also a single-institution study, which limits its generalizability. Whether a larger series of patients with more power would have shown more benefits is unknown.

## Conclusions

In patients with diffuse coronary disease, CE is a safe and feasible technique for a select group of patients with excellent mid-term survival rates and graft patency rates. CE produces better overall results when performed on the LAD, and grafted over with the LIMA. Similar outcomes are obtained with both on-pump and off-pump surgery. For a select group of patients, coronary endarterectomy (CE) offers an alternative choice of coronary artery reconstruction and complete coronary revascularization.

## Abbreviations

CABG: Coronary artery bypass grafting
CE: Coronary endarterectomy
CPB: Cardiopulmonary bypass
CTA: Computer tomography angiogram
EF: Ejection fraction
LIMA: Left internal mammary artey
NYHA: New York Heart Association
PI: Pulsation index
